# Management of Febrile Urinary Tract Infection With or Without Bacteraemia in Children: A French Case-Control Retrospective Study

**DOI:** 10.3389/fped.2020.00237

**Published:** 2020-05-28

**Authors:** Caroline Goeller, Marie Desmarest, Aurélie Garraffo, Stéphane Bonacorsi, Jean Gaschignard

**Affiliations:** ^1^Service de Pédiatrie Générale et de Maladies Infectieuses, Hôpital Universitaire Robert-Debré, APHP, Paris, France; ^2^Service des Urgences Pédiatriques, Hôpital Universitaire Robert Debré, APHP, Paris, France; ^3^Service de Microbiologie, Hôpital Universitaire Robert Debré, APHP, Paris, France; ^4^Université Paris Diderot, Sorbonne Paris Cité, Paris, France

**Keywords:** bacteraemia, *Escherichia coli*, pediatric care, urinary tract infection in infants, 3rd generation cephalosporin

## Abstract

**Background:** Febrile urinary tract infections (FUTIs) are common among children, and are associated with a bacteraemia between 4 and 7% of cases. No data is available concerning the management of children with a bacteraemic FUTI.

**Objectives:** To compare the antibiotic treatment (parenteral and total duration) among children with bacteraemic and non-bacteraemic FUTIs, and the mean hospital length of stay (LOS); to describe clinical, microbiological and imaging features of children with bacteraemic and non-bacteraemic FUTIs and observed management modifications when the blood culture was positive.

**Methods:** A retrospective case-control study between 2009 and 2015 at Robert Debré's Pediatric Emergency Department (Paris, France). Children with a bacteraemic FUTI were included and matched for age and sex with two children with a non-bacteraemic FUTI.

**Results:** We included 50 children with a bacteraemic FUTI matched to 100 children with a non-bacterameic FUTI. The mean duration of parenteral antibiotics was longer for bacteraemic children (6.7 *vs*. 4.0 days, *p* < 0.001) but this difference was only significant in children > 28 days-old. The mean total duration of antibiotic was similar (11.3 *vs*. 11.6 days, *p* = 0.61). The mean LOS was longer for bacteraemic children (5.1 *vs*. 2.0 days, *p* < 0.001) but this difference was only significant in children > 28 days-old. A positive blood culture changed the management in 66% of patients. Clinical features at presentation were comparable. Bacteraemic patients had a higher procalcitonin (*p* = 0.006) and C-reactive protein (*p* = 0.01), lower mean lymphocyte count (*p* < 0.001).

**Conclusions:** A bacteraemic FUTI in children induced a longer duration of parenteral antibiotic treatment, a longer hospitalization in children > 28 days-old, and a modification of management for 66% of patients.

## Keys Notes

- Adult guidelines recommend that a positive blood culture should not change the management of a febrile urinary tract infection (FUTI), as bacteraemia does not modify the outcome. No such recommendation exists for children.
- We showed that a positive blood culture changed the management of a FUTI in 66% of cases.
- A positive blood culture was associated with a longer parenteral treatment and hospital stay in children > 28 days-old. It was not associated with a longer total antibiotic treatment duration or a worse outcome.

## Background

Febrile urinary tract infections (FUTIs) are common in children. Eight percent of girls and 2% of boys aged <8 years old are concerned by FUTIs (1, 2). The risk of bacteraemia during a FUTI is age-dependent (3): it is estimated between 4 and 6% for children aged ≤ 1-year-old (4) to 7% for infants aged ≤ 3 months old (5, 6).

The French guidelines for management of adults with a FUTI do not recommend to perform blood culture (BC) (7) since bacteraemia does not modify prognosis, choice nor duration of antibiotic treatment (8–11). BC is recommended in adults in three cases: (1) uncertain diagnosis in uncomplicated FUTIs; (2) FUTIs complicated by a severe sepsis or septic shock; (3) FUTI requiring an urologic drainage (7). Those recommendations do not differ for pregnant woman (12). In children, guidelines for the diagnosis and treatment of FUTIs exist in many developed countries (2, 13–17). However, no advice is available concerning the clinical management of children with a FUTI associated with a bacteraemia.

Some authors stress that the lack of recommendations is responsible for varied clinical practices regarding the management of children with bacteraemic FUTIs (4). Some studies suggest that bacteraemic FUTI may not need to be treated differently than non-bacteraemic FUTI (4, 18–20) since their outcome is comparable.

In 2007, the French Agency for the Safety of Health Products (AFSSAPS) recommended a BC for children hospitalized for a FUTI. However, no recommendation concerned the management of these (15). In 2012, BC was no longer mentioned in the diagnostic strategy of FUTIs in children (1). In 2014, the French Group for Pediatric Infectious Diseases (GPIP) recommended performing a BC in two cases: (1) serious forms of FUTIs; (2) at-risk patients (<3 months or of pre-exiting uropathy) (16). None of the recent guideline on FUTIs advises against obtaining BC in this setting.

If BC are still often performed in pediatric emergency departments, do their results influence clinical practices? Can they help for the diagnosis? Can they guide antibiotics' duration? Do bacteraemic and non-bacteraemic FUTIs have different clinical, biological or radiological presentations?

The primary outcome of this study was to compare the parenteral antibiotic treatment duration among children with bacteraemic and non-bacteraemic FUTIs. The secondary outcomes were: (1) to compare the total antibiotic treatment duration; (2) to compare the mean hospital length of stay (LOS); (3) to describe the management modifications induced by a positive BC; (4) to compare the clinical and microbiological features, imaging and clinical evolution of children with bacteraemic and non-bacteraemic FUTIs.

## Methods

### Study Design and Setting

We performed a retrospective case-control observational study at the Pediatric Emergency Department (PED) of the Robert Debré University Hospital (Paris, France) from January 1st, 2009 to December 31st, 2015. Two non-bacteraemic children were randomly matched for age, gender and year of consultation with each bacteraemic child.

### Patient Identification and Inclusion Criteria

FUTIs were identified by an ICD-10 (International Classification of Diseases) discharged diagnosis of “pyelonephritis.” Bacteraemic FUTI were identified using an existing microbiological database. Children with non-bacteraemic FUTIs were pre-selected according to their age, gender and year of consultation and selected if the BC was negative. Children were excluded if they had a history of previous FUTI, urologic comorbidities, no BC drawn, or if fever at home or in the PED was not clearly mentioned in the medical record. A FUTI was confirmed if the urine culture (UC) contained ≥ 10^5^ colony-forming unit (cfu)/mL of a pathogenic organism (15). If more than one organism was identified in the UC, the child was included only if one of the bacteria was ≥ 10^5^ cfu/mL and prevailed over the others (difference of >10^2^ cfu/mL) and with existence of clinical arguments for a FUTI.

Decapitated urine samples were defined as the association of a sterile culture (after antibiotic treatment) with significant leukocytes count in the urine, and clinical arguments for a FUTI. Children with decapitated UC were included if the BC was positive for a compatible uropathogen, but were not included in the non-bacteraemic group.

### Data Collection

Medical records of bacteraemic and non-bacteraemic children were reviewed. Demographic, clinical and microbiological data, imaging (renal ultrasound—US, voiding cystourethrogram—VCUG), treatment, short-term and long-term clinical outcomes were collected. All the data were anonymised. Patients were considered ill-appearing if the medical record reported the child as being “lethargic,” “toxic,” “sick,” “ill-appearing” or “septic.” The following findings defined an abnormal renal US: hydronephrosis, duplicated renal collecting system, renal abscess, vesico-ureteral reflux (VUR), posterior urethral valves or ureteropelvic junction obstruction.

We defined as “modifications in the management of the children with a positive BC” the followings: (1) prescription of an additional BC; (2) prolongation of parenteral route for antibiotic treatment ≥ 4 days; (3) prescription of an additional antibiotic treatment.

### Statistical Analysis

For categorical variables, proportions were compared. For continuous ones, means with 95% confidence intervals (95% CI) or medians with interquartile values were compared. The Fisher's exact test was used to compare the proportions of categorical variables. Student's *t*-test was used to compare continuous variables. A two-tailed *p*-value < 0.05 was considered statistically significant.

### Ethical Considerations

We got Robert Debré institutional Review Board's approval for this study. A written informative letter was sent to families. Our database has been declared to the French National Commission for Information Technology and Civil Liberties (No. 2022430v0).

## Results

Fifty-seven children diagnosed with a bacteraemic FUTI were included between January 1st, 2009 and December 31st, 2015. Seven were excluded from the study: five had an urologic comorbidity (two posterior urethral valves, one duplicated renal collecting system, one primary hyperoxaluria type 1 with repeated kidney stones and renal complication and one ureteropelvic junction obstruction), one an uncertain diagnosis of FUTI and one child was transferred to another hospital. Between four children (in 2013 and 2014) and 12 (in 2011) were included each year. Children were between 12 days and 11 years old, with a median of 2 months [IQR = 1–4]. Thirty-six patients (72%) were male. One hundred non-bacteraemic patients were matched by age, sex, and year of consultation to the 50 bacteraemic children on a 2:1 basis.

### Clinical and Microbiological Features of Children With Bacteraemic and Non-bacteraemic FUTI

Demographic and clinical characteristics of children with a FUTI are listed in Table 1. Age, gender, and clinical presentation (among which fever duration and toxic-appearing) of children with positive and negative BC were comparable. On the other hand, children with positive BC presented more frequently a non-renal underlying medical condition (14 *vs*. 3%, *p* = 0.02). Comorbidities in the bacteraemic group were prematurity <34 weeks of gestation (WGA) (*N* = 4), Down syndrome (*N* = 1), congenital heart defect (*N* = 1) and sickle cell disease (*N* = 1). In the non-bacteraemic group, there was one child born prematurely at 29 WGA, one with sickle cell disease and one treated for an esophageal atresia.

**Table 1:** Clinical, microbiological, imaging characteristics and management of patients with FUTI.

	**Bacteraemic FUTIs** ***N* = 50**	**Non-bacteraemic FUTIs** ***N* = 100**	***p***
**Clinical data (mean)**			
Age in month, median [IQR]	2 [1-4]	2 [1-4]	0.81
Male gender (%)	36 (72)	72 (72)	1
Any comorbidity (%)	43 (86)	97 (97)	**0.02**
Heart rate, min^−1^ [95% CI]	175 [168–182)	171 [166–175]	0.27
Fever at admission, °C [95% CI]	38.1 [37.9–38.3]	38.3 [38.1–38.4]	0.24
Mean duration of fever, days [95% CI]	1.5 [1–1.9]	1.9 [1.5–2.3]	0.13
Toxic-appearing (%)	4 (8)	3 (3)	0.43
Antibiotics before consultation (%)	3 (6)	2 (2)	0.33
**Biological data (mean)**			
WBC, x10^9^/L [95% CI]	15.9 [13.9–17.8]	17.2 [10.5–23.9]	0.27
Neutrophil count, x10^9^/L [95% CI]	10.3 [8.2–12.3]	10.4 [9.3–11.5]	0.93
Lymphocyte count, x10^9^/L [95% CI]	4.3 [3.7–4.9]	6.2 [5.5–7.0]	**<0.001**
Platelet count, x10^9^/L [95% CI]	321 [281–362]	413 [388–438]	**<0.001**
C-reactive protein, mg/L [95% CI]	141 [118–165]	102 [87–117]	**0.013**
Procalcitonin, ng/mL [95% CI]	36.5 [15-28]	5 [0.9–9]	**0.006**
**Isolated bacteria in the urine culture**			
*Escherichia coli* (%)	45 (90)	98 (98)	**0.04**
*Proteus mirabilis* (%)	2 (4)	0	0.11
*Enterococcus faecalis* (%)	1 (2)	0	0.33
*Klebsiella pneumoniae* (%)	0	2 (2)	0.55
Decapitated sample (%)	2 (4)	0	0.11
**Radiological data**			
US performed (%)	50 (100)	99 (99)	1
US abnormal (%)	10 (20)	7 (7)	**0.03**
VCUG performed (%)	26 (52)	27 (27)	**0.004**
VCUG abnormal (%)	9 (18)	6 (6)	**0.04**
VUR (%)	8 (16)	6 (6)	0.07
≥ Grade III VUR (%)	5 (10)	3 (3)	0.12
**Clinical outcome (mean)**			
Fever duration, days [95% CI]	1.3 [1–1.6]	0.9 [0.2–1.7]	**0.025**
Recurent FUTI within 2 years (%)	5 (10)	3 (3)	0.12
Mortality	0	0	1
**Management of patients with FUTI (mean)**			
Hospitalized (%)	39 (78)	54 (54)	**0.004**
Length of stay, day [95% CI]	5.1 [4.1–6.1]	2 [1.4–2.7]	**<0.001**
Initial treatment by oral antibiotics (%)	1 (2)	1 (1)	1
Initial treatment by parenteral antibiotics (%)	49 (98)	99 (99)	1
Mean duration of parenteral antibiotics, days [95% CI]	6.7 [5.7–7.6]	4 [3.5–4.6]	**<0.001**
Switch to oral antibiotics (%)	35 (70)	89 (89)	**0.006**
Mean duration of switched oral antibiotics, days [95% CI]	4.7 [3.7–5.8]	7.5 [6.7–8.2]	**<0.001**
Total duration of antibiotic treatment, days [95% CI]	11.3 [10.5–12.1]	11.6 [10.8–12.4]	0.61
Total IV combination therapy (%)	42 (84)	56 (56)	**<0.001**
Before BC result (%)	37 (74)	56 (56)	**0.02**

*Values in bold are statistically significant result (<0.05). Nomenclature codes impose to write species names in italic*.

Patients with a bacteraemic FUTI presented a higher mean procalcitonin (PCT) (36.5 *vs*. 5 ng/mL, *p* = 0.006) and C-reactive protein (CRP) upon admission (141 *vs*. 102 mg/mL, *p* = 0.013). PCT had not been performed in seven children (14%) in the bacteraemic group and in 17 (17%) in the non-bacteraemic group (*p* = 0.81). The sole child with no CRP drawn had a PCT performed. Patients in the bacteraemic group had similar white blood cell count (15.9 *vs*. 17.2.10^9^/L, *p* = 0.27) but lower mean lymphocyte count (4.3 *vs*. 6.2.10^9^/L, *p* < 0.001) and lower mean platelet count (321 *vs*. 413.10^9^/L, *p* < 0.001) than in the non-bacteraemic group.

BC grew with *E. coli* in 46 cases (92%), *Proteus mirabilis* in 2 cases, *Enterococcus faecalis* and *Klebsiella pneumoniae* in 1 case each. The BC was positive after a mean of 1.3 days (95% CI [1.1–1.4]). *E. coli* was isolated from UC in the bacteraemic group in 45 cases (90%), followed by *P. mirabilis (N* = 2, 4%) and *E. faecalis* (*N* = 1, 2%) (Table 1). The 1-month-old girl with a FUTI due to *E. faecalis* had a grade IV VUR; the 4-month-old boy with *P. mirabilis* had a hydronephrosis on the renal US but no abnormality on the VCUG. Two (4%) urine samples were considered as decapitated in the bacteraemic group. There was no case in which blood and urine cultures grew different pathogens; moreover, bacteria from BC and UC always shared the same pattern on antibiotic susceptibility test.

Bacteria distribution was similar in the non-bacteraemic group: all UC identified *E. coli* except in two infants (2%) for which *K. pneumoniae* was isolated. These two infants had no abnormality on the renal US and no VCUG was performed.

One child with bacteraemic FUTI had an extended-spectrum beta-lactamase (ESBL) *E. coli*. There was no possibility of using oral drug for the oral switch for another child in the non-bacteraemic group (although it was not a ESBL *E. coli*).

### Imaging and Clinical Outcome of Children With Bacteraemic and Non-bacteraemic FUTI

Renal US were obtained for all bacteraemic and for 99 of 100 non-bacteraemic children. Abnormalities were more frequent in the bacteraemic group (20 *vs*. 7%, *p* = 0.03). In each group, one infant presented a renal abscess.

VCUG was performed more frequently in the bacteraemic group (52 *vs*. 27%, *p* = 0.004). Eight vesicoureteral reflux (VUR) and one hydronephrosis were found in the bacteraemic group (18%) *vs*. six VUR (6%) in the non-bacteraemic group (*p* = 0.04). VUR of grade III or IV affected five children (10%) in the bacteraemic group *vs*. three (3%) in the non-bacteraemic group (*p* = 0.12).

The mean duration of fever was longer in the bacteraemic group (1.3 *vs*. 0.9 days, *p* = 0.03).

No child died during this period. One 15-day-old boy diagnosed with a non-bacteraemic FUTI due to *Escherichia coli* developed a meningitis.

No significant difference was found between groups in regards to recurrent FUTI within 2 years: 10 *vs*. 3% (*p* = 0.12). Five infants with positive BC (aged 24 days, 1, 4, 8, and 16 months) presented another FUTI, respectively, 16, 1, 18, 9, and 2 months after the first episode. Three infants with negative BC aged 15–61 days had a recurrent FUTI after 1 and 2 months (missing data for the third child). *E. coli* was responsible for all these recurrent FUTIs except for the 15-day-old boy (*E. faecalis*). Seven of these eight infants with recurrent FUTI were diagnosed with a VUR on the VCUG and the last one with bilateral hydronephrosis.

### Management of FUTIs

#### Length of Stay (LOS)

Thirty-nine (78%) of the 50 children with a bacteraemic FUTIs were hospitalized *vs*. 54 of 100 (54%) non-bacteraemic ones (*p* = 0.004). Children were either hospitalized in a pediatric department or in the PED's short-term unit of hospitalization. Children never stayed more than 2 days in the PED's short-term unit of hospitalization: they were treated as outpatients in the PED after hospitalization (one visit per day) or hospitalized in a pediatric department to pursue their treatment.

Mean LOS was statistically different between patients with positive BC and those with negative BC: 5.1 (95% CI [4.1–6.1]) *vs*. 2 days [1.4–2.7], *p* < 0.001) (Table 1), but this difference only concerned children ≥ 28 days old (4.7 [3.5–5.9] *vs*. 1.3 [0.8–1.9], *p* < 0.001) (Table 2).

**Table 2:** Comparaison of clinical management of patients with FUTI depending of their age.

	**Age** **≤** **28 days**			**Age** **>** **28 days**		
	**BC + N = 10 (%)**	**BC – N = 13 (%)**	***p***	**BC + N = 40 (%)**	**BC – N = 87 (%)**	***p***
Hospitalized (%)	10 (100)	13 (100)	1	29 (73)	41 (47)	**0.01**
Length of stay, day [95% CI]	6.7 [5.1–8.3]	8.5 [5.5–11.4]	0.31	4.7 [3.5–5.9]	1.3 [0.8–1.9]	<0.001
Initial treatment by oral antibiotics (%)	0	0	1	1 (3)	1 (1)	0.54
Initial treatment by parenteral antibiotics (%)	10 (100)	13 (100)	1	39 (98)	86 (99)	1
Mean duration of parenteral antibiotics, days [95% CI]	10.2 [8.3–12.1]	9.7 [7.0–12.3]	0.76	5.8 [4.9–6.7]	3.5 [3.0–4.0]	<0.001
Switch to oral antibiotics (%)	2 (20)	5 (38)	0.67	33 (83)	84 (97)	0.01
Mean duration of switched oral antibiotics, days [95% CI]	0.6 [0–1.4]	3.0 [0–8.1]	0.17	5.8 [4.7–6.9]	7.9 [7.3–8.5]	<0.001
Total duration of antibiotic treatment, days [95% CI]	10.5 [8.7–12.3]	13.6 [8.4–18.7]	0.30	11.6 [10.7–12.4]	11.5 [10.9–12.1]	0.73
Total IV combination therapy (%)	10 (100)	12 (92)	1	32 (80)	44 (51)	0.001
Before BC result (%)	10 (100)	12 (92)	1	27 (88)	44 (51)	0.09

*Significant values in bold*.

#### Mean Duration of Parenteral Antibiotics

**Figure 1:**
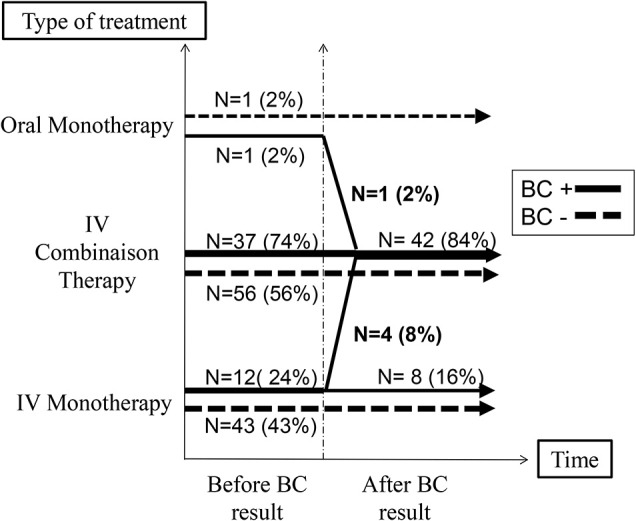
Antibiotic management according to the blood culture's result.

All patients were initially treated intravenously, except one in each group that received oral antibiotic treatment (Figure 1).

The mean duration of parenteral antibiotics was significantly longer for patients with bacteraemic FUTI (6.7 days, 95% CI [5.7–7.6] *vs*. 4 days [3.5–4.6] for non-bacteraemic children, *p* < 0.001) (Table 1), but this difference only concerned children ≥ 28 days old (5.8 days [4.9–6.7] *vs*. 3.5 [3.0–4.0], *p* < 0.001) (Table 2).

Bacteraemic patients were treated by a combination therapy more often than non-bacteraemic patients (84 *vs*. 56%, *p* < 0.001): a third-generation cephalosporin (most of the time, cefotaxime in the department of pediatric hospitalization or ceftriaxone in the PED's short-term unit of hospitalization and for outpatients who came once a day to the PED) and an aminoglycoside (either gentamicin or amikacin). Only one bacteraemic 3 month-old boy was first treated by amikacin for 2 days before an oral switch. Infants treated by a combination therapy were often younger with a median age of 1 month in both groups.

Eleven children (seven with positive BC and four with negative BC) received their treatment intra-muscularly at home after a short hospitalization with intravenous antibiotics (during 1.3 days, 95% CI [0.6–2.0] *vs*. 1.0 day [0.2–1.8], *p* = 0.60). They were all treated initially in a department of pediatric hospitalization and in the PED (where patients come once a day as outpatients) with an association of ceftriaxone/cefotaxime and aminoglycoside. The four non-bacteraemic children were all treated for a total of 10 days. In the bacteraemic group, three children were treated for 10 days, one 5 days and two 15 days; the last one was treated during 10 days with oral drug during 5 days (the only one to have switched to an oral treatment after having had intramuscular injections at home).

#### Mean Duration of Antibiotic Treatment

The mean duration of antibiotic treatment was similar for children with or without bacteraemia, respectively, 11.3 *vs*. 11.6 days (*p* = 0.61; Table 1). It was relatively stable between 2009 and 2015. All children were treated 15 days or less except four. An 11-year-old girl with a positive BC and a 20-day-old boy with negative BC were treated, respectively, for 24 and 42 days for a renal abscess. A 39-day-old boy was diagnosed with multilocular cyst and was treated initially for multiple renal abscesses for 30 days. Another 15-day-old boy had 21 days of IV antibiotics for a FUTI complicated by a meningitis due to the same *E. coli* strain.

Only 70% of bacteraemic children were switched to oral antibiotics *vs*. 89% of non-bacteraemic ones (*p* = 0.006). The mean duration of oral antibiotics was longer in the non-bacteraemic group (7.5 *vs*. 4.7 days, *p* < 0.001; Table 1).

### Observed Treatment Modifications When the Blood Culture Is Positive

When the BC was found positive, the clinician changed his management for 66% of patients: (1) Another BC was performed in 42% of children; (2) Intravenous antibiotic treatment was prolonged for another mean of 3.9 days (95% CI [2.5–5.2]) in 40%, in addition to the 4 days recommended by local guidelines; (3) 10% received an additional antibiotic (aminoglycoside) (Figure 1).

## Discussion

We have conducted a retrospective study on a 7-year period and compared 50 children with bacteraemic FUTI to 100 children with non-bacteraemic FUTI matched by age, sex, and year of consultation. Our study has shown that a positive BC was associated with a longer parenteral treatment and hospital stay in children ≥ 28 days old. A bacteraemia was also significantly associated with a higher CRP, a higher PCT and a larger prescription of VCUG. There was a trend to identify more VUR of grade III to V in the bacteraemic group. On the opposite, bacteraemia was not associated with a longer total antibiotic treatment duration nor a higher risk of recurrent FUTI in the 2 years following the first episode.

In our study, a positive BC was associated with a longer hospitalization (5.1 *vs*. 2 days; *p* < 0.001) and a longer duration of parenteral antibiotic treatment (6.7 *vs*. 4 days; *p* < 0.001) and a subgroup analysis showed this longer LOS concerned only children ≥ 28 days old. Our results were similar to Schroeder *et al* and to Roman *et al* who found, respectively, a mean duration of parenteral antibiotics of 7.8 and 6.8 days, and a mean LOS of 7.4 and 6.5 days (4, 18). Schroeder et al. (18) had excluded patients if they had received prolonged parenteral treatment for other reasons such as a meningitis or an osteomyelitis. We did not exclude the unique patient with meningitis in our study. As Roman et al. (4) we found that the distribution of LOS was similar to the duration of parenteral antibiotics, just a bit shorter. This can be explained by the management of children with parenteral antibiotics as outpatients in the PED and by the treatment of 10 other children treated intra-muscularly at home after a few days of hospitalization, which shortened the LOS.

We found no difference between children with bacteraemic and non-bacteraemic FUTI on the clinical presentation in the emergency department. In particular, bacteraemic children did not appear more toxic. While being not well-appearing was a risk factor for bacteraemia in 29–90 day-old infants in Hernandez-Bou's study (21). The clinical appearance of children with and without bacteremia was also indistinguishable in Hoberman's study (20).

In our study, CRP and PCT were significantly higher in the bacteraemic group, in accordance with another pediatric study (21).

*E. coli* was the predominant isolated bacteria in our study (92% of BC in the bacteraemic group and 98% of UC in the non-bacteraemic), in accordance with the literature (5, 15, 18, 20).

Unlike Pitetti and Choi's study (6), our patients with bacteraemic FUTI were more likely to have an abnormal renal US: 20 *vs*. 7% (*p* = 0.03). We found eight children (16%) with a VUR in the bacteraemic group and only six (6%) in the non-bacteraemic group (*p* = 0.07). Some studies report as high as 35% of VUR identified after a first FUTI, most of them are low grade VUR (5, 16, 17). The low proportion of patients who underwent VCUG (36%) in our study may account for this difference. Three children with a grade II VUR, one with grade III and three with grade IV were found in infants with normal renal US results. Our result (three cases of 37 VCUG performed in the normal renal US group, 8%) was quite similar to the 10% of low grade VUR found by Ismaili *et al* in infants with normal renal US (5).

Eight of the 150 included children (5%) had a recurrent FUTI between 1 and 18 months after the initial episode; however, some patients may have consulted elsewhere for other episodes of FUTI; we could not access this information. Schroeder et al. (18) found 2.4 % children who had a relapse in the 2 first months. Roman et al. (4) found 3.6% bacteraemic infants and 4.6% non-bacteraemic who had a recurrent FUTI within 30 days. Ismaili et al. (5) found 14% of recurrence within 8.3 +/– 7 months, but half of the patients had congenital abnormalities of the kidney and/or of the urinary tract.

Our study presented several limitations. First, some data were missing owing to the retrospective nature of our study. Second, the method for urine collection could not always be specified. Urine sample were often collected by clean bag, and some secondarily confirmed by urethral catheterization. No child had urine collected by suprapubic puncture, as this is not a practice in our hospital. Urine collection method was also unknown in 24.7% of the time in Schroeder's study (18). New recommendations tend to collect urine sample by other means than clean bag: transurethral catheterization, suprapubic aspiration or sample collected during the voiding (16). To limit the bias of contaminated sampled, we defined FUTI as a febrile infection with a UC containing a pathogenic organism with ≥ 10^5^ cfu/mL, more stringent than the AAP criteria of ≥ 5.10^4^ cfu/mL when the sample is collected by transurethral catheterization (4, 6, 21).

Our study was not designed to recommend or not to perform a BC in children with FUTI. But it appears that this BC is not without consequence in term of choice of treatment for the clinician. Although our study like others seems to show a similar evolution between bacteraemic and non-bacteraemic FUTI (6, 20, 22–24) and bacteraemic children were not more “toxic-appearing” than non-bacteraemic ones.

In our study, BC enabled the clinician to identify the causative pathogen of the FUTI when the UC was sterilized by an early antibiotic treatment. This concerned only two cases (4%), amongst which one infant younger than 3 months-old. This usefulness of BC is also mentioned in other papers (8, 25).

## Conclusion

Our study has shown that, without recommendation, clinician treats differently bacteraemic and non-bacteraemic FUTIs: a positive BC was associated with a longer parenteral treatment and hospital stay in children ≥ 28 days-old. It was not associated with a longer total antibiotic treatment duration, a worse outcome or a higher risk of recurrent FUTI in the 2 years following the first episode. A positive BC changed the clinical management of 66% of patients. Bacteraemia in FUTI was associated with higher CRP and PCT, lower platelet and lymphocyte counts, and a large prescription of VCUG. A multicentre prospective study will be useful to define the place of BC in children with FUTI and to standardize the management of these bacteraemic infections.

## Data Availability Statement

The datasets generated for this study are available on request to the corresponding author.

## Ethics Statement

The studies involving human participants were reviewed and approved by Robert Debré institutional Review Board's (Number 2017/379) and French National Commission for Information Technology and Civil Liberties (No. 2022430v0). Written informed consent to participate in this study was provided by the participants' legal guardian/next of kin.

## Author Contributions

CG, SB, AG, and JG contributed to the design of the study. CG and MD collected the data. CG and JG analyzed the data and mainly contributed to the writing of the manuscript. All authors revised the manuscript.

## Conflict of Interest

The authors declare that the research was conducted in the absence of any commercial or financial relationships that could be construed as a potential conflict of interest.
